# Emodin promotes fibroblast apoptosis and prevents epidural fibrosis through PERK pathway in rats

**DOI:** 10.1186/s13018-019-1357-9

**Published:** 2019-10-10

**Authors:** Guirun Xiong, Hui Chen, Qi Wan, Jihang Dai, Yu Sun, Jingcheng Wang, Xiaolei Li

**Affiliations:** Department of Orthopedics, Clinical Medical College of Yangzhou University, Orthopaedic Institute, Northern Jiangsu People’s Hospital, Yangzhou, 225001 China

**Keywords:** Emodin, Fibroblast apoptosis, PERK, Epidural fibrosis

## Abstract

**Background:**

Laminectomy is usually classed as a common orthopedic surgery, but postoperative epidural fibrosis often leads to less-than-desirable clinical outcomes. As demonstrated by prior studies, emodin (EMO) exerts an anti-fibrotic effect. Here, we carried out investigation into the inhibitory effect created by EMO application on epidural fibrosis after laminectomy in rats.

**Methods:**

The paper conducts a series of experiment. In vitro, we observed the effect of EMO on fibroblasts by Cell Counting Kit-8 (CCK-8) assay. Apoptosis of fibroblasts induced by EMO was detected by western blot, TUNEL assay, and flow cytometry. The results revealed that EMO was capable of inducing fibroblast apoptosis, and the proteins of PERK pathway also changed accordingly. In vivo, the effect of EMO on epidural fibrosis in 12 male Sprague-Dawley rats was observed by histological staining.

**Results:**

CCK-8 assay indicated that EMO was effective in reducing fibroblast viability in a time- and a dose-dependent manner. TUNEL assay and flow cytometry analysis have demonstrated that the apoptotic rate of fibroblasts increased as the EMO concentration rose. Western blot analysis proved that EMO promoted the relative expression of p-perk and p-eIF2α and that the expression of its downstream proteins CHOP and GRP78 was also enhanced. The expression of apoptotic protein Bax and cleaved PARP was upregulated, whereas the expression of anti-apoptotic protein Bcl-2 was downregulated. In addition, histological and immunohistochemical analysis demonstrated that EMO functioned to inhibit epidural fibrosis and increase GRP78 expression in fibrous tissue by promoting apoptosis of fibroblasts.

**Conclusions:**

EMO could have inhibitory effect on epidural fibrosis in a concentration-dependent manner. The potential mechanism might be through PERK signaling pathway to promote fibroblast apoptosis. It has a possibility to be taken as a novel method for the treatment of epidural fibrosis.

## Background

Laminectomy is extensively applied to the treatment of spinal stenosis and spinal neoplasms [1], postoperative fibrosis is classified as a common complication of the surgery, and epidural fibrosis is capable of compressing nerve tissue in the spine and causing a variety of different symptoms [2], including significant dysfunction and recurrent radiculopathy. Plenty of measures have been taken to prevent postoperative fibrosis. At the present time, enabled by the constantly improving surgical techniques, clinicians have taken a range of measures to prevent epidural fibrosis, for instance, the application of innovative biological materials [3] and local or systemic application of medicine [4]. Despite this, the treatment effect of complications after lumbar spine surgery remains far from satisfactory [5].

As indicated by studies, fibroblast proliferation is the major cause of postoperative fibrosis [6]. Recently, it has been discovered that fibroblast apoptosis is also increasing during the process of decline in fibroblast proliferation [7]. This is believed to present an alternative way to inhibit fibrosis by promoting apoptosis [8], despite the exact mechanism still in the exploratory stage.

Emodin (1,3,8-trihydroxy-6-methylanthraquinone EMO) is a natural anthraquinone derivative that is present as a major component of various herbs, such as rhubarb, *Polygonum multiflorum*, and cuspidatum [9]. People regard these herbs as medicinal materials. Many investigators are paying increasing attention to the active ingredient (EMO) contained in these herbs. This compound exhibits various pharmacological benefits, like anti-viral, anti-bacterial, anti-allergic, anti-diabetic, immunosuppressive, and hepatoprotective activities [9]. Recently, multiple studies have reported that EMO is identified as a potential anti-fibrotic agent [10]. Prior studies have confirmed that EMO is safe for the treatment of renal fibrosis and capable to prevent postoperative intra-abdominal adhesion formation [11], suggesting that it might be also useful in the treatment of epidural fibrosis.

As a significant organelle, endoplasmic reticulum (ER) performs various functions, such as protein folding, modification, and processing as well as the formation, assembly, and transportation of new peptide chains [12]. When folding errors accumulated to a certain extent, a series of reactions would occur. If folding errors are left unresolved, apoptosis will occur through the C/EBP homologous protein (CHOP) pathway [13]. Recently, a number of studies have demonstrated that apoptosis induced by ER stress is beneficial for the treatment of fibrosis diseases [14].

Consequently, it is of interest to determine whether EMO is capable of inducing apoptosis induced by ER to reduce epidural fibrosis. It is hoped to provide a fresh idea for the treatment of epidural fibrosis.

## Materials and methods

### Reagent

Emodin (molecular formula: C_15_H_10_O_5_ EMO) [9] was purchased from Shanghai Aladdin Biochemical Technology Co., Ltd. The purity of EMO is 95%.

### Cell culture and EMO treatment

Human fibroblasts were sourced from ScienCell Research Laboratories (Shanghai, China). Then, the cells were cultured in DMEM (Invitrogen, CA, USA) which contains 15% fetal bovine serum (FBS, Gibco, USA) and 1% penicillin/streptomycin (Gibco, CA, USA) under 5% CO_2_ at 37 °C. Fibroblasts between passages 4 and 6 were involved in all experiments. The cells were transferred into various dishes overnight. After reaching 60–70% density, the cells were washed with phosphate-buffered saline. Subsequently, various concentrations (0, 5, 10, 20 μg/ml) of EMO were applied to treating the fibroblast.

### Cell viability assay

The cell viability of fibroblasts treated with EMO was detected by Cell Counting Kit-8 (CCK-8) (Dojindo, Tokyo, Japan). The cells at exponential stage were transferred into 96-well plates. When the cell density reached 60–70%, EMO of varying concentrations was added to each well for a period of 24 h. Then, the cells were treated with 10 μl CCK-8 solution well for another 2 h. The absorbance at 450 nm was measured by microplate absorbance reader (TECAN). In the same way, the cells with EMO (10 μg/ml) were treated for differing lengths of time (0–72 h). Cell survival rate was calculated according to the instruction book.

### Flow cytometry analysis of fibroblast apoptosis

Fibroblasts were cultured into six-well plates, before being incubated for 24 h. Three wells were assigned as the control group, and another three wells were set as the EMO-treated group. After the EMO-treated group was treated with 10 μg/ml EMO for 24 h, all cells were collected, prior to being washed with 4 °C PBS buffer for three times. The cells with 1 ml 1× binding buffer were resuspended, and 100 μl cell suspension was transferred to the tube. Then, 5 μl PI and 5 μl FITC Annexin V were added to the tubes. After the addition of 400 μl 1× binding buffer and incubation by FITC Annexin V and PI for 15 min at room temperature away from light, the mixture was detected by flow cytometry.

### Western blot analysis

After the EMO of varying concentrations was treated, all cells were collected. Resuspended fibroblasts were lysed by RIPA buffer (Beyotime, Shanghai, China) for 15 min. The protein concentration was measured by using BCA Protein Assay Kit (Beyotime, Shanghai, China). Forty micrograms of protein per well was used for western blot. After being soaked in the blocking buffer at room temperature for a 2-h spell, the PVDF membranes (Millipore, Bedford, MA) were incubated with primary and secondary antibodies successively in line with the instructions. The primary antibodies used were anti-cleaved-poly ADP-ribose polymerase (cleaved PARP), anti-Bax, anti-Bcl-2, anti-78-kDa glucose-regulated protein (GRP78), anti-CHOP, anti-PERK, anti-phospho-PERK (P-PERK), anti-eukaryotic translation initiation factor 2α (eIF2α), anti-phospho-eIF2α(P-eIF2α), and anti-GAPDH antibodies (CST, Beverly, MA, USA). The secondary antibodies involved were the anti-mouse or anti-rabbit IgG (CST, Beverly, MA, USA).

### TUNEL assay staining in fibroblasts

Fibroblasts were seeded into six-well plates, with a glass slide contained in each well for 24 h. After treatment of 10 μg/ml EMO for 24 h, the fibroblasts were fixed by 4% paraformaldehyde at room temperature for 15 min. The TUNEL staining (KeyGEN, Nanjing, China) procedures were developed based on the manufacturer’s instructions. After brief steps of staining, fluorescence microscopy was employed for detection of the apoptotic fibroblasts.

### Animal laminectomy model and local application of EMO

The study was granted approval from the Animal Ethics Committee of Yangzhou University. Twelve male 250–280 g SD male rats were split into three groups on a random basis, namely saline group, 50 mg/ml emo group, and 100 mg/ml emo group. Based on previous studies [15, 16], rats were anesthetized by intraperitoneal injection of 1% pentobarbital sodium (40 mg/kg), and we performed laminectomy model to remove L1-L2. Subsequently, topical 1 × 1 cm gauze containing the corresponding (0/50/100 mg/kg) concentration of EMO covered the wound for 5 min, prior to the wounds being rinsed with saline and sutured in layers carefully**.**

### Histological analysis

Four weeks later, after anesthesia, all rats were perfused with 4% paraformaldehyde. The spine of rats after laminectomy was collected by groups, and the specimen was immersed in formalin for 3 days before decalcification with ethylenediamine tetraacetic acid (EDTA) for 1 month. Successive 4-μm sections were obtained through the surgical vertebra. Hematoxylin-eosin (HE) and Masson’s trichrome staining were used to evaluate the degree of epidural fibrosis. The images of epidural fibrosis, scar adhesion, and collagen synthesis were observed by optical microscopy at the magnification of × 40. The fibroblast counting was calculated by three fields (100 × 100 mm each) in the sites of epidural defect at the magnification of × 100.

### Immunohistochemical staining

After denitrification and rehydration, these sections were subjected to pretreatment with sodium citrate to activate their antigenicity. Endogenous peroxidase is blocked by 3% hydrogen peroxide. The sections of epidural fibrosis tissue were incubated with anti-GRP78 at room temperature for 1 h and then incubated with anti-rabbit IgG at room temperature for 2 h. Then, the sections were stained by using DAB kit and counterstained by using hematoxylin. Finally, sections were observed under a light microscope.

### Statistical analysis

The data was analyzed with SPSS statistical 19.0 software. All of our data were presented as mean ± standard deviation. Independent Student’s *t* test was conducted to draw comparison between groups. *P* value < 0.05 was introduced to define statistical significance.

## Results

### EMO inhibits cell viability and induces apoptosis in fibroblasts

**Fig. 1:**
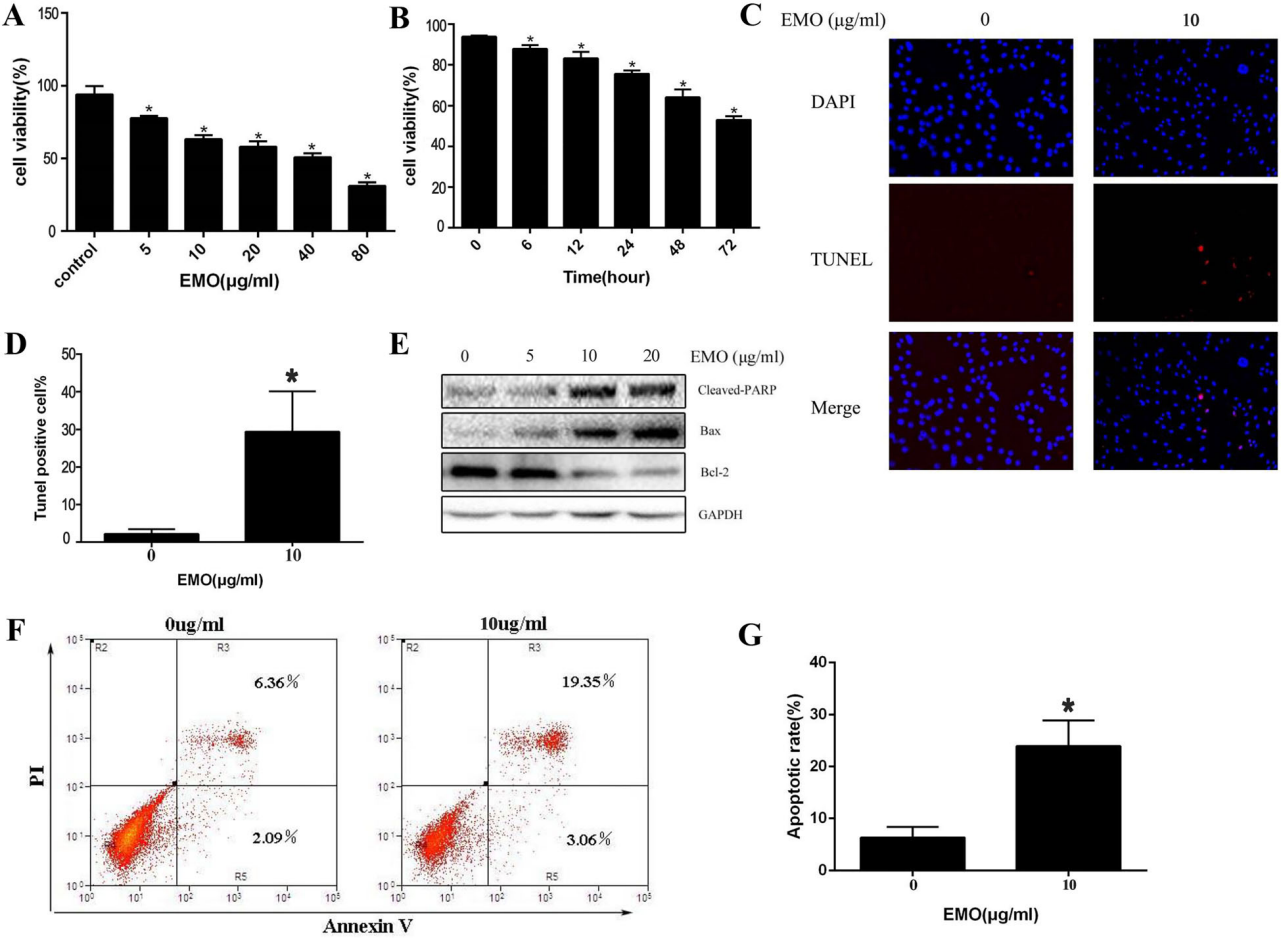
**a** After fibroblast treated with various concentrations of EMO for 24 h, the CCK-8 assays showed that EMO inhibited the cell viability in a concentration dependent. **b** Following the 10 μg/ml EMO-treated fibroblasts, the CCK-8 assays showed that EMO could inhibit the cell viability in a time-dependent manner. **c** After treatment of fibroblasts with EMO of 10 μg/ml for 24 h. TUNEL staining was performed to detect the effect of EMO in promoting apoptosis. All nuclei of fibroblasts were stained blue by DAPI, and the nuclei of TUNEL-positive fibroblasts were red. **d** We picked three fields to calculate the percentage of apoptotic fibroblasts in each group. *P < 0.05 versus the control group. **e** Western blot analysis showed that the expression of apoptosis-related proteins Bcl-2, Bax, and cleaved PARP changed with the increasing of EMO concentration. GAPDH was used as a control. **f** Annexin V-FITC/PI double labeling was performed to detect fibroblast apoptosis after 10 μg/ml EMO treated for 24 h. **g** The histogram presented the apoptotic rate after the treatment with EMO by statistical method. The result was repeated for three times. *P < 0.05 versus the control group

To ascertain whether EMO could induce fibroblast apoptosis, the cells were treated with varying concentrations of EMO for 24 h. Besides, the fibroblasts with EMO (10 μg/ml) were treated for different lengths of time (0–72 h). Then, the CCK-8 assay was performed to detect the effect of EMO on the cell viability. As revealed in Fig. 1a, b, EMO plays a role in inhibiting fibroblast viability in a dose- and time-dependent manner. In order to validate the effect of EMO on fibroblast apoptosis, we performed morphological examinations (TUNEL assay). As shown in Fig. 1c, d, the control group was found to have few TUNEL-positive cells; however, the TUNEL-positive cells were observed to have a significant increase in the EMO-treated group**.** Besides, as EMO concentration was on the rise; western blot (Fig. 1e) analysis showed that the expression of pro-apoptotic markers was upregulated, such as cleaved PARP and Bax. By contrast, the expression of anti-apoptotic marker Bcl-2 was in decline. As revealed by the Annexin V-FITC/PI double labeling (Fig. 1f, g), the apoptosis rate of fibroblast was increased after EMO treatment. In summary, the above results evidenced that EMO is effective in inducing fibroblast apoptosis significantly.

### ER stress-mediated apoptosis induced by EMO

**Fig. 2:**
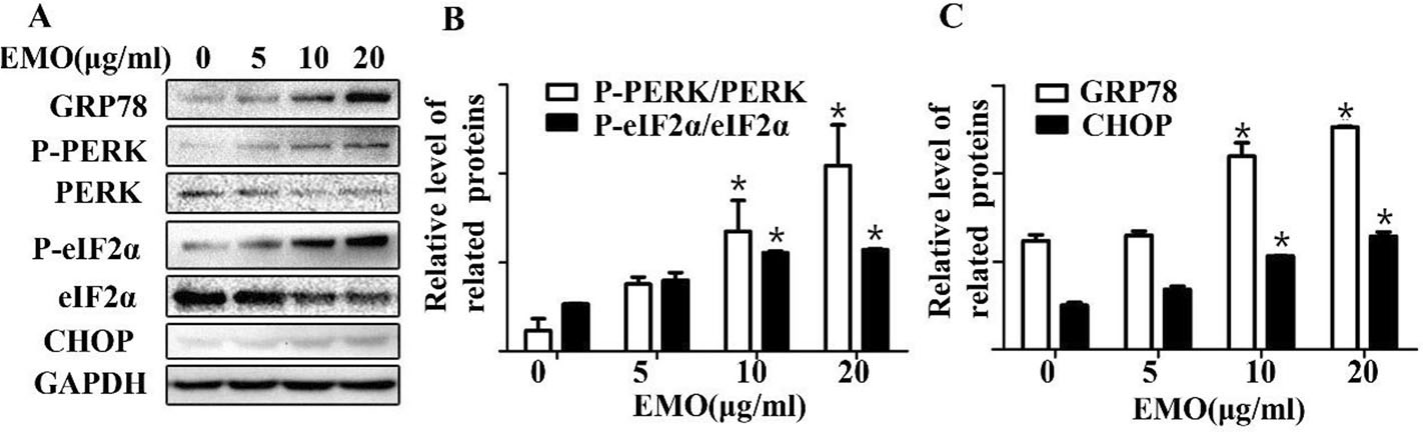
**a** EMO induces protein-related changes in ER stress pathway in a dose-dependent manner, including GRP78, P-PERK, PERK, P-eIF2a, eIF2a, and CHOP, which were detected by western blot following the treatment of 0, 5, 10, and 20 μg/ml EMO for 24 h. GAPDH was set as a loading control. **b** The histogram shows the band intensity ratio of P-PERK/PERK and P-eIF2a/eIF2a. The result was repeated for three times. *P < 0.05 versus the control group. **c** The expression of GRP78 and CHOP relative to GAPDH was shown as a histogram. The result was repeated for three times. *P < 0.05 versus the control group

In order to figure out the molecular mechanism of EMO on fibroblast apoptosis, the cells with four concentrations of EMO were treated, and then, the expression of ER stress pathway-related proteins was detected by performing western blot analysis. Figure 2a demonstrates that EMO promoted the expression of GRP78, p-PERK, and p-eIF2α. Then, the expression of two ER stress pathway proteins (GRP78 and CHOP) (Fig. 2b) was analyzed, which led to the discovery that the protein expression was upregulated when the EMO concentrations were increased. Then, detection was made of a classic pathway of ER stress relative protein (PERK, elF2α, P-PERK, P-elF2α) (Fig. 2c), and the ratios of P-PERK/PERK and P-elF2α/elF2α showed a noticeable upsurge with the concentration of EMO on the increase. All the results indicated that EMO induces fibroblast apoptosis via the upregulation of PERK signal pathway.

### EMO reduced epidural fibrosis in rats

**Fig. 3:**
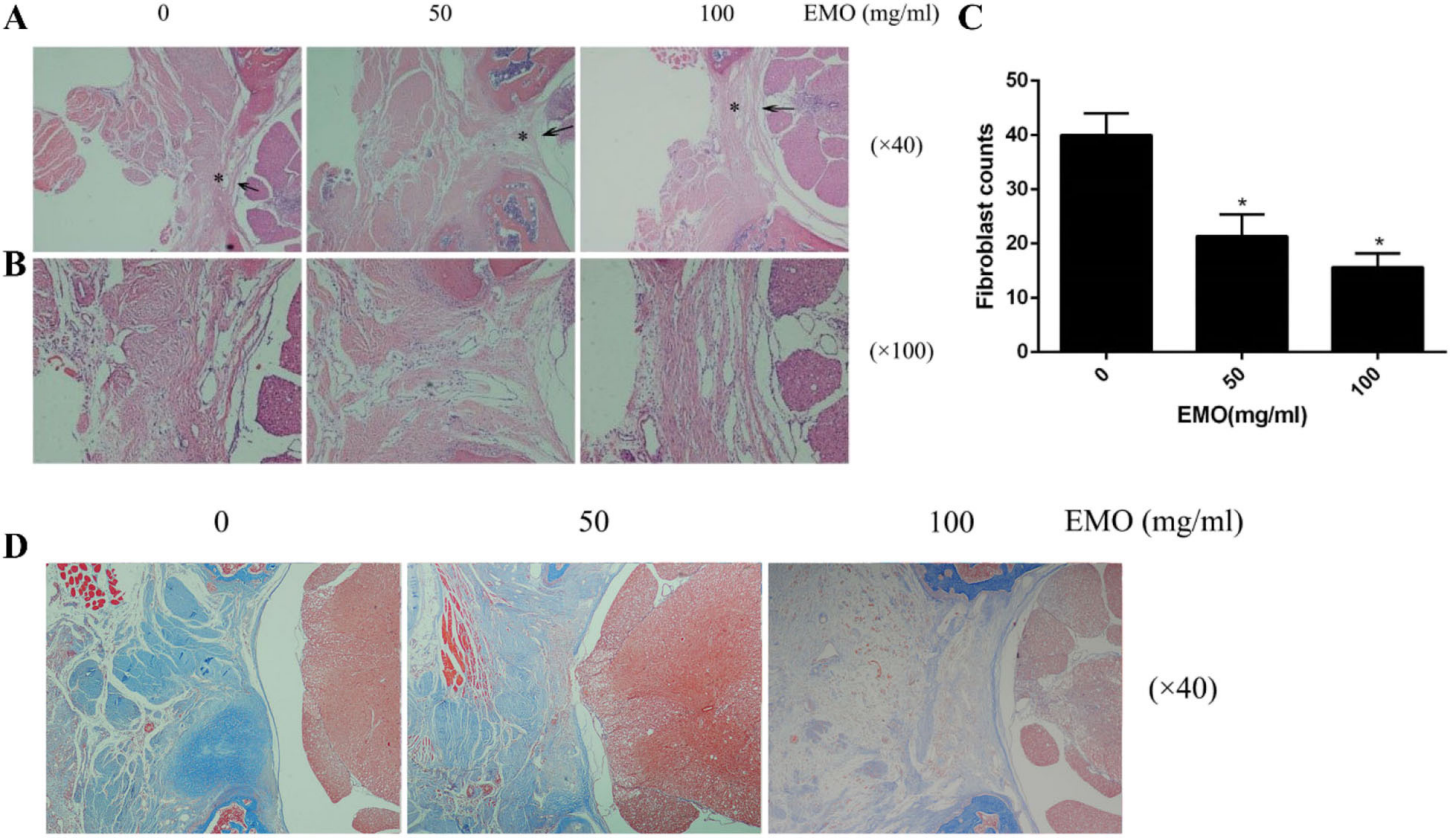
EMO prevented epidural fibrosis and inhibited fibroblast proliferation in rats. **a** Histological analysis images of the laminectomy operation areas treated with 100 mg/ml and 50 mg/ml EMO, and saline. The scar tissues were marked by asterisk, the magnification was × 40, and the fibrous tissue of the EMO group was significantly reduced in a concentration-dependent manner compared with the control group. These sections were stained by hematoxylin and eosin (HE). **b** The number of fibroblasts was reduced as the EMO concentration increased. The magnification was × 100. **c** We picked three fields to count fibroblast number from every section. *P < 0.05 versus the control group. **d** Histological analysis of the effect of EMO on collagen density in rat after laminectomy. With the increase of EMO concentration, the local collagen fiber density decreased

Figure 3a reveals that the control group epidural fibrosis was more severe as compared to the EMO-treated group. In addition, with the increase in EMO concentration, the degree of epidural fibrosis was in decline incrementally. The HE staining images (× 100) show that the number of fibroblasts in the EMO-treated groups was less than that in the control group (Fig. 3b, c). The Masson staining images (Fig. 3d) show that the density of collagen was higher in the control group than in the EMO-treated group. Moreover, the density of collagen decreased as its concentration rose.

### EMO upregulated the expression of GRP78 in the epidural scar tissue in rats

**Fig. 4:**
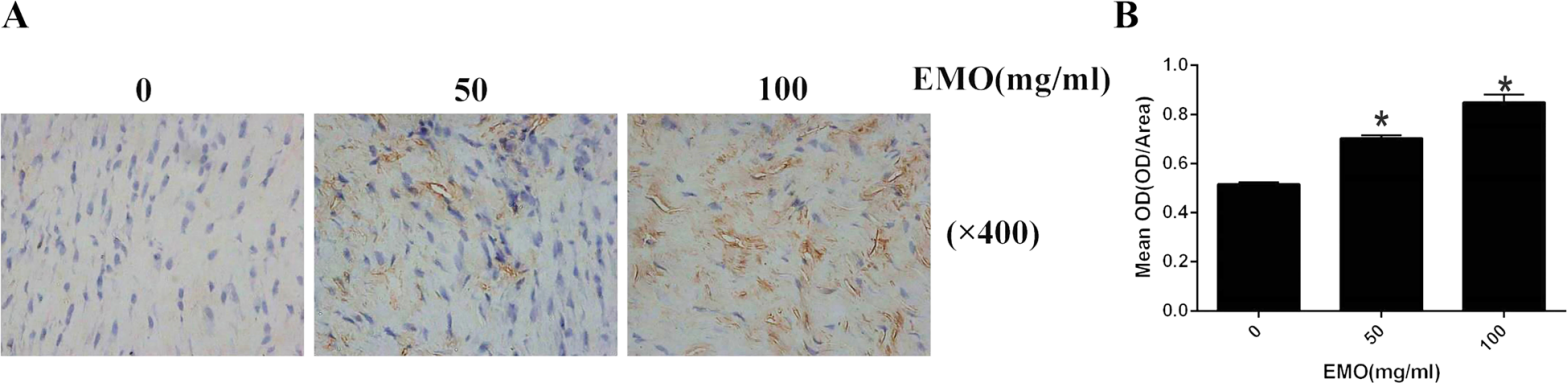
EMO upregulated the expression of GRP78 in the epidural scar tissue in rats. **a** Immunohistochemical analysis of the expression of GRP78 in epidural scar tissue after treatment with saline and 50 and 100 mg/kg EMO. **b** The histogram shows the optical analysis of immunohistochemistry, and the results were repeated for three times

Figure 4a, b demonstrates that the expression of GRP78 was enhanced with the rising level of EMO concentrations in the epidural scar tissue. GRP78 expression in the 100 mg/kg EMO-treated group was noticeably increased as compared with the control group. In previous studies [15], it was demonstrated that GRP78 increased notably after ER stress activation. All these results suggested that ER stress signaling plays an essential role in EMO-induced reduction of epidural fibrosis.

## Discussion

Epidural fibrosis is understood as an important cause of failed back surgery syndrome (FBSS) [17]. Although the molecular mechanism remains unclear, plenty of studies have confirmed that epidural fibrosis is associated with fibroblast proliferation [1]. In previous studies, various measures have been taken to prevent adhesion formation, for instance, surgical improvement and application of biological materials [3] as well as application of topical or systemic medication [15]. However, the results are still less than satisfactory.

In previous studies [18–20], it was discovered that EMO is capable of exerting various effects, such as anti-inflammatory effects [18], cardiovascular protection [19], and prevention of liver fibrosis [20] and renal fibrosis [11]. Recently, it was found out that EMO could induce apoptosis in resistant acute myeloid leukemia cells [21]. Thus, an inference can be made that EMO could also induce apoptosis of fibroblasts.

According to previous study, apoptosis is a programmed cell death and represents a crucial means of maintaining cell homeostasis [22]. Recently, a discovery was made that inducing fibroblast apoptosis might be beneficial for treating fibrosis diseases [23]. Besides, it was known that ER stress is a classical pathway for apoptosis [24]. Once ER stress signaling is activated, the expression of CHOP, an important protein of ER stress, is certain to increase, and the expression of ER molecular chaperone (GRP78) would be upregulated. Subsequently, the expression of PERK and pathway proteins was also changed, which led to the occurrence of apoptosis [25].

In this experiment, CCK-8 assay was involved to detect the cell viability of fibroblasts after EMO was treated, which led to the finding that EMO could inhibit cell viability at a certain concentration and time dependence. Subsequently, western blot detected the expression of cleaved PARP and Bax, and Bcl-2, annexin V FITC/PI double labeling and TUNEL assay were performed and the results showed that EMO could promote fibroblast apoptosis. In order to ascertain whether or not ER stress was activated after being EMO treated, the expression of CHOP and GRP78 was measured, and the expression of CHOP and GRP78 was found to be increased, which indicated that EMO activates ER stress. Subsequently, the ER stress pathway proteins were detected by conducting western blot analysis, which led to the finding that the PERK signal pathway was activated. Therefore, it can be concluded that EMO is possible to induce fibroblast apoptosis through ER stress.

Afterwards, EMO of differing concentrations was applied to the laminectomy rat model topically. The selected EMO concentration was based on previous studies [26], and the effect of EMO on epidural fibrosis was subjected to evaluation by histological observation and fibroblast counting. These evaluation indexes suggested that topical application of EMO could alleviate epidural fibrosis. The immunohistochemistry staining revealed that expression of GRP78 was increased after topical application of EMO. Combined with the effect of EMO on fibroblast apoptosis in vitro, all results indicated that the anti-proliferation and anti-fibrosis effects of EMO on epidural fibrosis were achieved via ER stress.

In this study, no toxic or side effects of EMO were observed on treated rats. It has been known that EMO has anti-inflammatory [27] and anti-tumor effects [28]. However, emodin could also lead to hepatotoxicity, kidney toxicity, and reproductive toxicity, particularly in high doses and with long-term use [9]. Therefore, the minimum effective concentration was ought to be adopted to treat rats to ensure security. Lentiviral or PERK signaling pathway inhibitors were not involved in this study to block signaling pathways. More experiments might be necessary to further validate the relationship between emodin and PERK signaling pathway.

## Conclusion

In summary, it can be concluded that the use of appropriate concentrations of EMO is effective in reducing epidural fibrosis after laminectomy in rats. This effect is possible to promote apoptosis achieved through activation of the ER stress signaling pathway to enhance apoptosis. This is hoped to provide a new way to reduce epidural fibrosis after laminectomy.

## Data Availability

The datasets supporting the conclusions of this article are included within the article and its supplementary materials.

## Abbreviations

CCK-8: Cell Counting Kit-8
DAB: Diaminobenzidine
EDTA: Ethylenediamine tetraacetic acid
EMO: Emodin
ER: Endoplasmic reticulum
GRP78: 78-kDa glucose-regulated protein
HE: Hematoxylin and eosin
PBS: Phosphate-buffered saline
SD: Sprague-Dawley
